# Exosomal miR-130b-3p Promotes Progression and Tubular Formation Through Targeting PTEN in Oral Squamous Cell Carcinoma

**DOI:** 10.3389/fcell.2021.616306

**Published:** 2021-03-22

**Authors:** Wei Yan, Yuping Wang, Yong Chen, Yanjun Guo, Qiang Li, Xiaotong Wei

**Affiliations:** ^1^Department of Oral and Maxillofacial Surgery, Cangzhou Central Hospital, Cangzhou, China; ^2^Department of Stomatology of Shennongju Hospital, Huanghua, China

**Keywords:** oral squamous cell carcinoma, exosome, miR-130b-3p, progression, Pten, tubular formation

## Abstract

Oral squamous cell carcinoma (OSCC), accounting for two-thirds of head and neck cancer, is characterized by poor prognosis and a high rate of mortality. Exosomes have emerged as potential molecule-shuttle in intercellular communication, thereby regulating the physiological processes of recipient cells. To date, the effect of exosomal microRNAs (miRNAs) on the progression of OSCC has not been fully investigated. In this study, we found that the protein, but not mRNA expression of Phosphatase and tensin homolog deleted on chromosome 10 (PTEN) was decreased in OSCC. The results revealed that miR-130b-3p was an important negative regulator for PTEN expression. Additionally, overexpression and knockdown of miR-130b-3p enhanced and inhibited angiogenesis in human umbilical vein endothelial cells (HUVECs), respectively. Also, miR-130b-3p was transferred by exosomes to HUVECs and then promoted angiogenesis and inhibit the expression of PTEN. Furthermore, exosomal miR-130b-3p derived from OSCC cells promoted tumor growth and blood vessel formation in the xenograft mice model. Taken together, we demonstrated that exosome-mediated miR-130b-3p promoted progression and tubular formation in OSCC *in vitro* and *in vivo*. These results would provide new insight into exploring biomarkers and effective therapeutic strategies for OSCC.

## Introduction

Head and neck cancer (HNC) has emerged as a significant health issue worldwide, with over 450,000 new diagnosed patients per year (Torre et al., 2015), of which more than 95% of malignant tumors are squamous cell carcinomas, including oral squamous cell carcinoma (OSCC; Chinn and Myers, 2015). OSCC is characterized by a high rate of morbidity and mortality and poor prognosis, with a 5-year survival rate of 60%, but ranging from 10 to 82% due to age, race, tumor location, and stage (Chinn et al., 2013). Also, cigarette consumption is thought as the riskiest factor for OSCC, which significantly elevates the risk of developing OSCC (Hashibe et al., 2009). Although the incidence of OSCC has reduced in the last decade with the development of diagnosis and treatment, the outcomes are still not satisfied thanks to only 5% improvement in overall survival rate in the last two decades (Chinn and Myers, 2015). Thus, investigating the underlying mechanism and exploring new biomarkers and therapeutic strategies would be an essential and urgent need for patients with OSCC.

Exosomes are nano-sized (30–150 nm in diameter) lipid bilayer membrane vesicles (Zeringer et al., 2015; Barile et al., 2017). Exosomes are released from diverse cell types into the extracellular space and then internalized by recipient cells. Several biological essential molecules, including microRNAs (miRNAs), enzymes, long non-coding RNAs, are delivered via exosomes, thereby regulating cellular activities of recipient cells (Zhang et al., 2016). As such, in recent years, exosomes have been demonstrated to be a promising intercellular communication mediator to transport functional molecules. Increasing evidence has revealed that exosome-delivered molecules play an essential role in regulating cancer, including tumor development, progression, angiogenesis, recurrence, metastasis, and chemoresistance (Zhang et al., 2015). Of which, miRNAs are a group of well-studied exosome-shuttled molecules in biological and pathological processes of various cancer types (Melo et al., 2014; Thind and Wilson, 2016).

As an anti-tumor factor, Phosphatase and tensin homolog deleted on chromosome 10 (PTEN) is found to be mutated in several tumor types (Salmena et al., 2008), that associated with the critical role of PTEN in tumor cell biology, including angiogenesis (Wen et al., 2001). Angiogenesis is a formative process of new blood vessels based on existing vascular tissues, with high invasion ability of cancer cells and increased microvessel density (Folkman, 1971). Angiogenetic activity is closely associated with various pathological processes, including cancer, inflammation, as well as ischemic-related diseases (Jiang et al., 2000). To date, numerous studies suggest that PTEN plays an essential role in angiogenesis. For example, PTEN/Phosphatidylinositol 3−kinase (PI3K) is an essential signaling pathway to modulate tumor angiogenesis and normal vascular development (Hamada et al., 2005). PTEN is also involved in several tumor-associated angiogeneses, such as glioblastomas (Hsu et al., 1996), prostate cancer (Giri and Ittmann, 1999), and pancreatic cancer (Ma et al., 2009).

Based on these previous studies, the objective of this study thus was to investigate whether exosome-delivered miRNA can regulate the progression in OSCC and related mechanisms.

## Materials and Methods

### Ethics Statement

All patients recruited in the present study were informed, and written consent was provided by all patients. All experimental designs and protocols were approved by the Ethics Committee of Cangzhou Central Hospital. Meanwhile, all animal-involved experiments were approved by the Institutional Animal Care and Use Committee of Cangzhou Central Hospital.

### Tissue Preparation

Oral squamous cell carcinoma tissues and adjacent control tissues were collected from patients who underwent surgical procedures at Cangzhou Central Hospital from 2015 to 2017. All patients were provided written informed consent. The basic information about twenty patients was summarized in Table 1. The inclusion criteria of patients with OSCC included: (1) having complete medical records; (2) no medical history of other cancers; (3) being diagnosed with OSCC by pathological assessment; and (4) not receiving radiotherapy or chemotherapy treatment before operation. The exclusion criteria were as follows: (1) having multiple diseases, such as cardiovascular diseases, respiratory disease, and gastrointestinal disease; (2) having physical disabilities; (3) surgery-intolerant; and (4) received other treatments prior to the operation. After sample collecting, all tissues were histologically assessed by experienced pathologists. Tissues were immediately placed in liquid nitrogen and then transferred to the laboratory and stored at −80°C.

**TABLE 1:** Patient characteristics.

	***N***	**Percentage**
All cases	20	
**Age(years)**		
<60	5	25
≥60	15	75
**Gender**		
Male	13	65
Female	7	35
**UICC stage**		
I–II	6	30
III–IV	14	70
**Grade**		
Low	8	40
High	12	60
**Tumor size(cm)**		
<3	6	30
≥3	14	70
**Location of the tumors**		
Border of tongue	10	50
Alveolar mucosa/gingiva/retromolar area	6	30
Floor of mouth/ventral tongue	2	10
Buccal mucosa/buccal sulcus	2	10
**Therapy**		
Radiotherapy	11	55
Chemotherapy	9	45
Surgery (R0-resection)	20	100
**Median overall survival**		
Months	25.1	
Range	1–231	

### Cell Culture

The human OSCC cell line (OECM1) (CN: SCC180) and human umbilical vein endothelial cells (HUVECs) (CN: 20005N) were purchased from Sigma-Aldrich (St. Louis, MO, United States). Control cells were isolated from adjacent control tissues. All cells were cultured in DMEM containing 10% fetal bovine serum (Gibco, United States) and antibiotic solution (Sigma) in a humidified incubator (37°C, 5% CO_2_) (Thermo Fisher Scientific, United States). Certificates of OECM1cell line authentication were provided as Supplementary Material 1. Additionally, because the OECM1 cell line was not comprehensively validated due to unexpected COVID lockdown, the findings of this study should be interpreted or applied with caution.

### qRT-PCR

Total RNAs were isolated from cells (1 × 10^6^) or exosomes (1 μg/ml) by using TRIzol Reagent (Invitrogen, United States) according to the manufacturer’s instructions. The quality control of total RNAs was determined by spectrophotometry at 260/280/230 nm by using NanoDrop 1000 Spectrophotometer (Thermo Fisher Scientific, United States). The integrity of RNA was assessed by using RNA StdSens analysis kit (Bio-Rad, United States) according to the manufacturer’s instructions. First-strand cDNAs were synthesized using the Maxima Reverse Transcriptase kit (Thermo Fisher Scientific, United States) according to the manufacturer’s instructions. miRNA expression was detected by using Taqman miRNA probes (Applied Biosystems, United States). PCR reactions were performed on ABI StepOne Real-Time PCR System (Applied Biosystems, United States) according to the manufacturer’s instructions and recommended reaction conditions. Primers for qRT-PCR were designed with Primer Express 3.0 software (Applied Biosystems). The primer amplification efficiencies were 0.9153–0.9331(slope: −3.543 to −3.493) (Supplementary Material 2). The primers used in this study were as following: PTEN: 5′-GAGGGATAAA ACACCATG-3′ (forward) and 5′-AGGGGTAGGATGTGAACCAGTA-3′ (reverse); GAPDH: 5′-AGAAGGCTGGGGCTCATTTG-3′ (forward) and 5′-AGGGGCCATCCACAGTCTTC-3′ (reverse); miR-130b-3p: 5′-GCCGCCAGTGCAATGATGAA-3′ (forward) and 5′-A GTGCAGGGTCCGAGG-3′ (reverse); U6: 5′-CTCGCTTCGGCAGCACA-3′ (forward) and 5′-AACGCTTCACGAATTTGCGT-3′ (reverse). The PTEN and miR-130b-3p expression were normalized to GAPDH and U6, respectively. All PCR reactions were carried out in triplicate, and PCR data were analyzed by using the 2^–Δ^ ^Δ^ ^*Ct*^ approach (Livak and Schmittgen, 2001).

### Western Blotting

Total protein was isolated from cells (1 × 10^6^) or exosomes (1 μg/ml) by using cell lysis buffer (Thermo Fisher Scientific, United States). Western blotting assay was performed according to a previous study (Chen et al., 2014). The antibodies against PTEN (1:1000; Catalog #: sc-7974), GAPDH (1:2000; Catalog #: sc-47724), TSG101 (1:1,000; Catalog #: sc-7964), Alix (1:1,000; Catalog #: sc-53540), CD63 (1:1,000; Catalog #: sc-5275), and secondary antibody goat anti-mouse IgG-HRP (1:2,000; Catalog #: sc-2005) were purchased from Santa Cruz Biotechnology (Shanghai, China). The protein expressions were quantified by using ImageJ (Rasband, 2011).

### Cell Transfection

miR-130b-3p mimics and inhibitors were obtained from Applied Biological Materials (abm Inc., New York, NY, United States). The sequence of miR-130b-3p mimics and inhibitors were as follows: 5′-CAGUGCAAUGAUGAAAGGGCAU-3′ and 5′-UGCCAACCUUGCAAGCCGAAG-3′, respectively. Small interfering RNAs (siRNAs) (Catalog #: sc-36752) and negative control (cat. no. Sc-36869 were purchased from Santa Cruz Biotechnology (Shanghai, China). Cells (1 × 10^6^) were plated in six-well plates and then were transfected with 100 nM mimics/inhibitors or 2 μl siRNA/negative control by using the Lipofectamine 3000 (Invitrogen, United States) according to the manufacturer’s instructions. The cells were used for subsequent experiments 24 h after transfection.

### Exosome Isolation and Identification

Exosomes were isolated from the serum (5 ml) and cell culture medium by using ExoQuick ULTRA (System Biosciences, United States) according to the manufacturer’s instructions. The morphology and diameter size distribution of exosomes were assessed by FEI Tecnai T20 transmission electron microscopy (TEM; FEI Company, United States) according to the previous study (Li et al., 2015). The number of exosomes was determined by applying the BCA protein assay (Thermo Fisher Scientific, United States) (Soares Martins et al., 2018).

### Luciferase Reporter Assay

Wild-type and mutated binding sequence of miR-130b-3p in 3’UTR of PTEN were cloned into T-Vector pMD20 (TaKaRa Biotechnology, Japan; Catalog #: 3270) and then subcloned to luciferase reporter p-MIR-REPORT vector (Thermo Fisher Scientific, United States; Catalog #: AM5795). Cells were plated in six-well plates and co-transfected with 50 ng wide-type or mutant reporter constructs, miR-130b-3p mimics or negative control, and 10 ng Renilla plasmid. Luciferase reporter assay was performed by using the Lipofectamine 3000 (Invitrogen, United States) according to the manufacturer’s instructions. pRL-Renilla luciferase control reporter vectors were used as a transfection control (Promega, United States). After 24 h transfection, cells were lysed and relative luciferase activity was determined by Luciferase Reporter Assay Kit (BioVision, Inc., United States). Firefly luciferase activity was normalized to Renilla, and relative luciferase activity was expressed as the ratio of firefly/renilla.

### Cell Proliferation and Migration Assay

According to previous studies, cell proliferation and migration assays were performed in HUVECs (1 × 10^6^) (Sarver et al., 2010; Du et al., 2012; Kinoshita et al., 2012). In brief, HUVECs were cultured in 50 μM Edu (RiboBio, China) for 12 h and then fixed with 4% paraformaldehyde for 30 min. Then, HUVECs were washed with PBS solution and permeabilized by using PBS (0.3% Triton X-100). Next, cells were sequentially treated with Apollo staining solution (20 min), NaCl/Pi (10 min), and DAPI staining solution (10 min; 1:2,000) at room temperature. For migration assay, HUVECs were suspended in a serum-free medium. HUVECs were placed in the upper compartment of Transwell Chamber (6.5 mm) with 8-μm pore-size polycarbonate membranes., DMEM medium supplemented with 10% FBS was placed in the lower compartment. After 6 h incubation, cells that migrated to the lower compartment were fixed with ethanol (90%) for 15 min and stained with 0.1% crystal violet solution at room temperature. Images were taken by using a photomicroscope (200×). Migrated cells were quantified by randomly selecting five fields per chamber.

### Ring Formation Assay

Ring Formation assay in HUVECs was conducted based on the previous protocol (Okamoto et al., 2014; Chuang et al., 2015). In brief, 100 μl Matrigel (BD Biosciences, United States) was added to a 24-well plate and incubated at 37°C for 30 min before the experiment. HUVECs (1 × 10^6^) were first treated with exosomes and were resuspended with FBS-free DMEM. Six hours later, The formation of the capillary-like structure was monitored under a light microscope. Images were taken by using a photomicroscope (200×). The branch-point of the tubes was quantified by randomly selecting five fields per well.

### Tumor Xenografts Mice Model

Male nude C57BL/6 mice (Jackson Laboratory, 6–7 weeks old; *n* = 5 for each group) were housed in standard conditions and allowed free access to food and water. OECM1 cells (1 × 10^6^ cells) transfected with miR-130b-3p-overexpressing lentivirus (miR-OE), miR-130b-3p-knockdown lentivirus (miR-KD), the combination of miR-KD and OECM1-exo (10 μg) (miR-KD + exo), and TSG101-knockdown lentivirus (TSG101-KD) were suspended in 200 μl PBS solution and then were subcutaneously injected into each flank of mice. The tumor size of each mouse was recorded weekly. Mice were sacrificed after seven weeks. Tumor volume was measured and calculated using the formula V = (L × W^2^) × 0.5 (where L is the tumor length and W is the tumor width) (Faustino-Rocha et al., 2013).

### Immunohistochemistry

Tumors collected from OSCC tissues or xenograft mice were fixed in 4% paraformaldehyde and then incubated in paraffin. After sectioning (4 μm in thickness), sections were placed at room temperature overnight to dry. Next, sections were dewaxed in xylene and rehydrated by using graded concentrations of ethanol. Subsequently, sections were incubated in citrate buffer solution (pH 6.0) and were placed in thermostatic water for antigen retrieval. Antibodies against PTEN (1:500; Catalog #: sc-7974) or CD31(1:1,000; Catalog #: sc-376764) (Santa Cruz Biotechnology, China) were used to incubated with sections overnight at 4°C. Sections were incubated with secondary antibody for 1h at room temperature and then incubated with streptavidin peroxidase and stained with 3,3′-diaminobenzidine chromogen (Santa Cruz Biotechnology, China). Images were taken by using a photomicroscope. Immune-positive cells were quantified by randomly selecting five sections.

### Statistical Analyses

Statistical analysis was performed using SPSS v.19.0 software (SPSS, IL, United States). Values were presented as mean ± SD obtained from three independent experiments. Differences between groups were analyzed with Student’s *t*-test. *^∗∗∗^p* < 0.001, *^∗∗^*p < 0.01, *^∗^*p < 0.05.

## Results

### PTEN Protein Expression Is Decreased in OSCC

**FIGURE 1:**
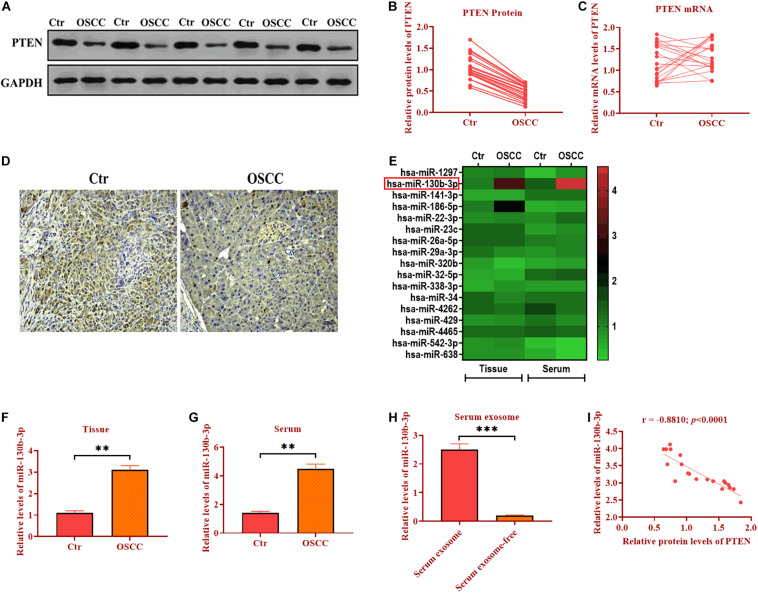
PTEN protein expression is decreased in OSCC. **(A,B)** Protein expression of PTEN in OSCC tissues and paired adjacent control tissues. **(C)** mRNA expression of PTEN in OSCC tissues and paired adjacent control tissues. **(D)** Protein expression of PTEN in OSCC tissues and paired adjacent control tissues, as determined by IHC assay. **(E)** The expression of seventeen candidate miRNAs in OSCC tissues and the serum of OSCC patients. **(F)** The expression of miR-130b-3p in OSCC tissues and paired adjacent control tissues. **(G)** The expression of miR-130b-3p in the serum of OSCC patients and control subjects. **(H)** The expression of miR-130b-3p in exosomes isolated from the serum of OSCC patients and control subjects. **(I)** The correlation between expression of PTEN and miR-130b-3p. ***p < 0.001, **p < 0.01.

In OSCC tissues, western blotting assay revealed that the protein level of PTEN was decreased (∼2 folds) compared with those of adjacent control tissues (Figures 1A,B). However, the mRNA expression of PTEN did not display the difference between OSCC and control tissues (Figure 1C), indicating that post-transcription may play a role in the regulation of PTEN in OSCC. Because only one endogenous control (GAPDH) was used for data analysis of qRT-PCR assay, these qRT-PCR results should be interpreted or used for future study with caution. Also, IHC assay was applied to assess the abundance of PTEN and the results showed that PTEN was significantly reduced in OSCC cells relative to control cells (Figure 1D). Together, PTEN may act as an anti-tumor factor in OSCC, and the downregulation of PTEN is associated with post-transcriptional regulation.

### Negative Correlation Between PTEN and miR-130b-3p in OSCC

MicroRNAss have been demonstrated to be essential post-transcriptional regulators of target gene expression (Obernosterer et al., 2006). Thus, we hypothesize that miRNAs may play a role in the regulation of PTEN expression. We then performed the bioinformatic analysis to predict the potential miRNAs targeting PTEN in OSCC by using TargetScan^1^ and miRanda.^2^ After measuring seventeen candidate miRNAs through qRT-PCR in another ongoing experiment (Figure 1E), we found miR-130b-3p was found to be upregulated in both OSCC tissues (∼3 folds) and the serum (∼3 folds) of OSCC patients compared with control tissues and serum (Figures 1F,G). Thus, miR-130b-3p was chosen for subsequential experiments. Also, miR-130b-3p was found to be highly enriched in exosomes derived from the serum of patients with OSCC (Figure 1H). In addition, the correlation analysis showed that the protein level of PTEN was negatively correlated with the expression of miR-130b-3p (Figure 1I). Collectively, miR-130b-3p may function as an oncogene targeting PTEN in OSCC.

### Serum-Derived Exosomes Deliver miR-130b-3p in OSCC

**FIGURE 2:**
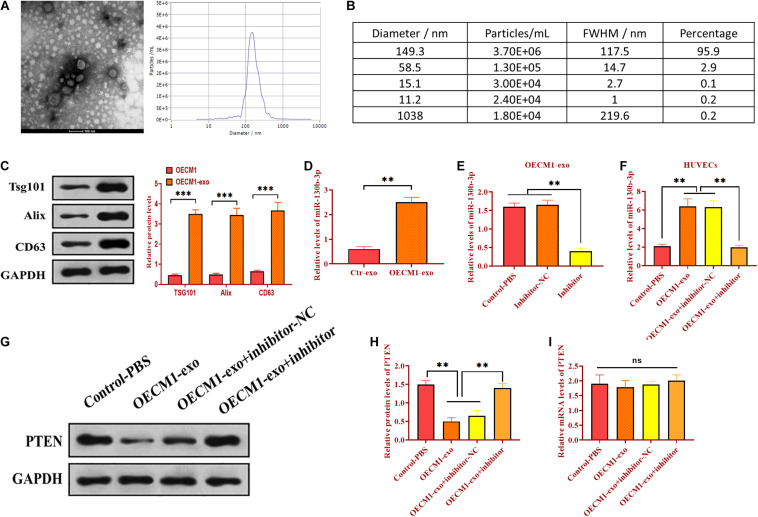
Exosomal miR-130b-3p in the serum reduces PTEN expression. **(A,B)** The morphology of exosomes and the exosome diameter size distribution, determined by transmission electron microscope. Scale bar = 100 nm. **(C)** The expressions of exosome markers Tsg101, Alix, and CD63. **(D)** The expression of miR-130b-3p in exosomes derived from control (Ctr-exo) and OECM1 (OECM1-exo) cells. **(E)** The expression of miR-130b-3p in OECM1-exo treated with miR-130b-3p inhibitor. **(F)** The expression of miR-130b-3p in HUVECs treated with OECM1-exo and the combination of OECM1-exo and miR-130b-3p inhibitor. **(G,H)** The protein expression of PTEN in HUVECs treated with OECM1-exo and the combination of OECM1-exo and miR-130b-3p inhibitor. **(I)** The mRNA expression of PTEN in HUVECs treated with OECM1-exo and the combination of OECM1-exo and miR-130b-3p inhibitor. **p < 0.01.

Exosomes have emerged as molecule-shuttle in intercellular communication, thereby regulating cellular activities in recipient cells (Simons and Raposo, 2009). We isolated exosomes from the serum of OSCC patients and evaluated the morphology and diameter size through TEM. As showed in Figures 2A,B, exosomes displayed a spherical shape, ∼149.3 nm (95.9%) in diameter. Exosomes were also verified by detecting exosomal markers TSG101, Alix, and CD63 (Figure 2C). Also, the expression of miR-130b-3p was higher in OECM1-derived exosomes (OECM1-exo) compared with exosomes secreted from control cells (Ctr-exo; Figure 2D), suggesting exosomes may carry miR-130b-3p in OSCC. To further verify this hypothesis, OECM1 cells were transfected with miR-130b-3p inhibitor, and the results showed that the level of miR-130b-3p was greatly reduced in OECM1-derived exosomes (Figure 2E). Next, we cocultured HUVECs with exosomes derived from OECM1 transfected with miR-130b-3p inhibitor negative control or inhibitor. We found that the expression of miR-130b-3p was elevated in HUVECs cocultured with OECM1-exo while was not affected in HUVECs treated with exosomes derived from miR-130b-3p inhibitor-treated OECM1 cells (Figure 2F). Accordingly, PTEN protein level was inhibited by OECM1-exo but not by OECM1-exo derived from OECM1 cells transfected with miR-130b-3p inhibitor (Figures 2G,H). Also, the mRNA level of PTEN was not affected in all groups (Figure 2I). Together, OECM1-exo was able to deliver miR-130b-3p to HUVECs to inhibit the protein expression of PTEN.

### Exosomal miR-130b-3p Targets PTEN

**FIGURE 3:**
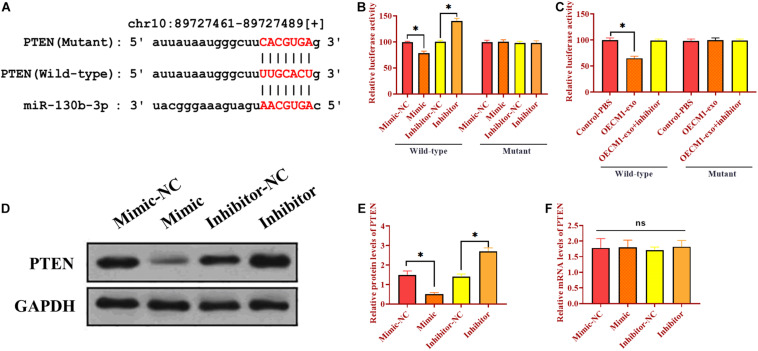
Exosomal miR-130b-3p directly targets PTEN. **(A)** Putative binding site of miR-130b-3p in 3’UTR of PTEN. **(B)** Relative luciferase activity in OECM1 cells transfected with luciferase reporter vector carrying wild-type or mutant binding site of miR-130b-3p in 3’UTR of PTEN and miR-130b-3p mimic or inhibitor. **(C)** Relative luciferase activity in OECM1 cells transfected with luciferase reporter vector carrying wild-type or mutant binding site of miR-130b-3p in 3’UTR of PTEN and OECM1-exo or the combination of OECM1-exo and miR-130b-3p inhibitor. **(D,E)** The protein expression of PTEN in HUVECs treated with miR-130b-3p mimic or inhibitor. **(F)** The mRNA expression of PTEN in HUVECs treated with miR-130b-3p mimic or inhibitor. *p < 0.05.

As mentioned, there was a negative correlation between PTEN and miR-130b-3p. We then determined whether miR-130b-3p could physically interact with PTEN via performing luciferase reporter assay. Through bioinformatics analysis, we obtained a putative binding site of miR-130b-3p in 3’UTR of PTEN (Figure 3A). Wild-type and mutant sequences of miR-130b-3p binding site were synthesized in 3’UTR of PTEN and then inserted into luciferase reporter plasmids. The modified luciferase plasmids and miR-130b-3p mimic or inhibitor and their respective negative controls were used to co-transfect with OECM1 cells. In OECM1 cells transfected with plasmids carrying wild-type 3’UTR of PTEN, relative luciferase activity was increased in cells transfected with miR-130b-3p inhibitor while decreased in those treated with miR-130b-3p mimic or OECM1-exo (Figure 3B and 3C). Meanwhile, relative luciferase activity did not change in OECM1 cells transfected with plasmids containing mutant 3’UTR of PTEN in all treatment groups (Figures 3B,C). Moreover, the protein level of PTEN was decreased in OECM1 cells transfected with miR-130b-3p mimic while increased in those treated with miR-130b-3p inhibitor (Figures 3D,E). However, upregulation and downregulation of miR-130b-3p both did not affect the mRNA expression of PTEN. Together, these results demonstrated that miR-130b-3p negatively regulates PTEN through directly targeting its 3’UTR (Figure 3F).

### Exosomes Derived From OECM1 Cells Promotes Angiogenesis

**FIGURE 4:**
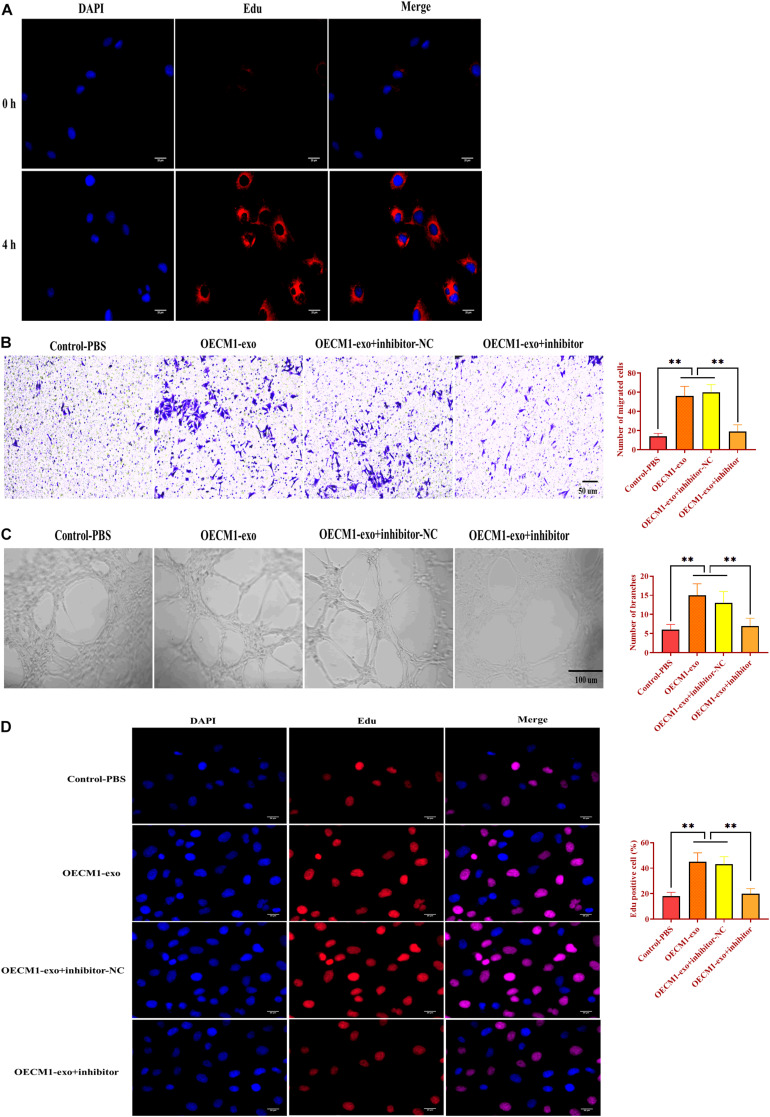
Exosomes derived from OECM1 cells promote angiogenesis. **(A)** Exosomes derived from OECM1 cells (OECM1-exo) were uptaken by HUVECs. Blue color indicated nuclei stained with DAPI and red color indicated exosomes stained with Edu. **(B)** Migration ability of HUVECs treated with OECM1-exo or the combination of OECM1-exo and miR-130b-3p inhibitor. **(C)** Ring formation ability of HUVECs treated with OECM1-exo or the combination of OECM1-exo and miR-130b-3p inhibitor. **(D)** Proliferation of HUVECs treated with OECM1-exo or the combination of OECM1-exo and miR-130b-3p inhibitor. ^∗∗^*p* < 0.01.

To investigate the function of OECM1-exo in angiogenesis, we cocultured HUVECs with OECM1-exo. By fluorescence assay, we found that OECM1-exo was successfully internalized by HUVECs (Figure 4A). Then, OECM1-exo was found to promote the abilities of migration and ring formation in HUVECs while such promotive effects were reversed by exosomes derived from OECM-1 cells transfected with miR-130b-3p inhibitor (Figures 4B,C). In addition, the same effect of OECM1-exo was also found in the proliferation of HUVECs (Figure 4D). Thus, these results suggest that exosomes derived from OECM1 cells could promote angiogenesis.

### MiR-130b-3p Is Essential to Angiogenesis

**FIGURE 5:**
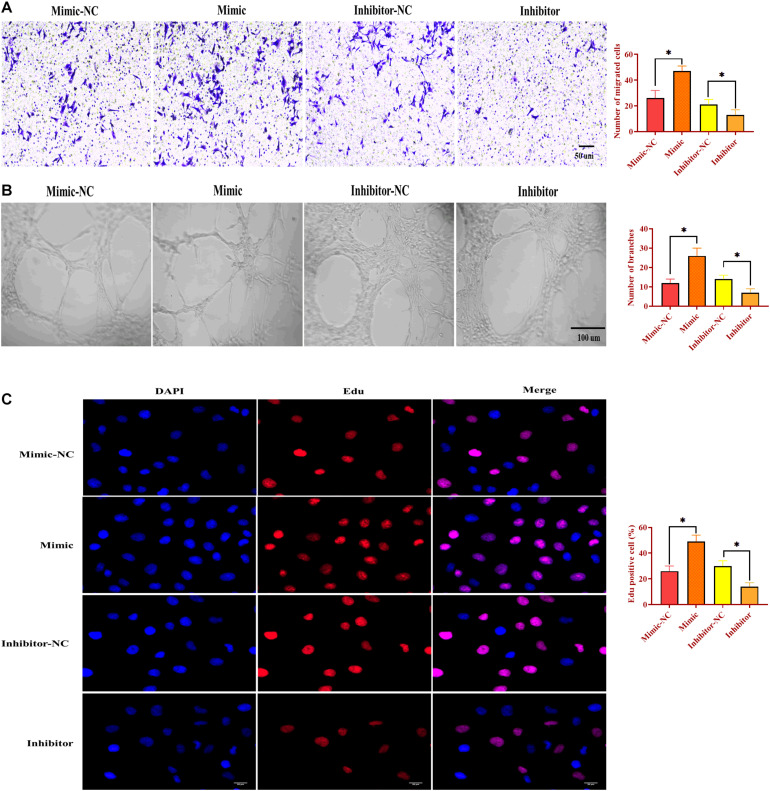
MiR-130b-3p is essential in angiogenesis in HUVECs. **(A)** Migration ability of HUVECs treated with miR-130b-3p mimic or inhibitor. **(B)** Ring formation ability of HUVECs treated with miR-130b-3p mimic or inhibitor. **(C)** Proliferation of HUVECs treated with miR-130b-3p mimic or inhibitor. ^∗^*p* < 0.05.

To determine the function of miR-130b-3p in angiogenesis, HUVECs were transfected with miR-130b-3p mimic or inhibitor. Similar to the effects of OECM1-exo, overexpression of miR-130b-3p promoted migrated cell rate, ring formation ability, and proliferation of HUVECs, which were reversed by the knockdown of miR-130b-3p (Figure 5). Thus, the results together indicate that miR-130b-3p is an essential mediator in the effect of OECM1-exo on the angiogenesis of HUVECs.

### Exosomal miR-130b-3p Promotes Tumor Growth *in vivo*

**FIGURE 6:**
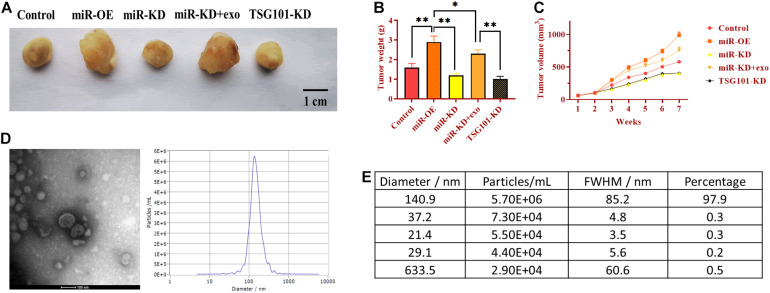
Exosomal miR-130b-3p promotes tumor growth *in vivo*. **(A)** Representative image of tumor tissues. **(B,C)** Tumor weight and volume of mice injected with OECM1 cells treated with miR-130b-3p-overexpressing lentivirus (miR-OE), miR-130b-3p-knockdown lentivirus (miR-KD), the combination of miR-KD and OECM1-exo, and TSG101-knockdown lentivirus (TSG101-KD), respectively. **(D,E)** The morphology of exosomes and the exosome diameter size distribution, determined by transmission electron microscope. Scale bar = 100 nm. **p < 0.01, *p < 0.05.

**FIGURE 7:**
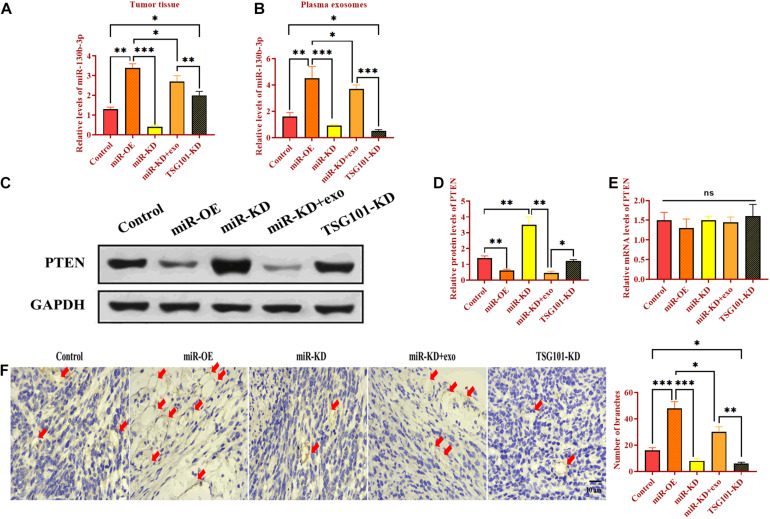
Exosomal miR-130b-3p promotes angiogenesis *in vivo*. **(A,B)** The expression of miR-130b-3p in tumor tissues and plasma exosomes in mice injected with OECM1 cells treated with miR-130b-3p-overexpressing lentivirus (miR-OE), miR-130b-3p-knockdown lentivirus (miR-KD), the combination of miR-KD and OECM1-exo, and TSG101-knockdown lentivirus (TSG101-KD), respectively. **(C–E)** The protein and mRNA expression of PTEN in mice injected with OECM1 cells treated with miR-130b-3p-overexpressing lentivirus (miR-OE), miR-130b-3p-knockdown lentivirus (miR-KD), the combination of miR-KD and OECM1-exo, and TSG101-knockdown lentivirus (TSG101-KD), respectively. **(F)** Tubular formation in tumor tissues was determined by IHC assay by using anti-CD31 antibody. ***p < 0.001, **p < 0.01, *p < 0.05.

To determine the role of exosomal miR-130b-3p in tumorigenesis and tubular formation *in vivo*, OECM1 cells transfected with miR-OE, miR-KD, the combination of miR-KD and OECM1-exo, and TSG101-KD were subcutaneously injected into nude mice. After sacrificing, tumor weight and diameter were decreased in mice treated with miR-KD and TSG101-KD while increased in those treated with miR-OE (Figures 6A–C). Notably, the addition of OECM1-exo reversed the effect of miR-KD in tumor growth (Figures 6A–C). Next, we isolated exosomes from the plasma of mice treated with different treatment. The morphology and characteristics of exosomes were evaluated by TEM (Figures 6D,E). The results showed that exosomes were ball-shape and mostly displayed ∼140.9 nm (97.9%) in diameter. As shown in Figures 7A,B, the expression of miR-130b-3p was found to be increased in both tumor tissues and the plasma of mice injected with miR-130b-3p-overexpressing cells while decreased in mice injected with miR-130b-3p-knockdown cells. OECM1-exo rescued the level of miR-130b-3p in mice treated with miR-KD. Also, TSG101-KD treatment increased miR-130b-3p level in tumor tissues while reduced in the plasma. Meanwhile, the protein expression of PTEN was negatively coorelated with the level of miR-130b-3p (Figures 7C,D). The mRNA expression of PTEN did not show the difference in all groups (Figure 7E). Finally, we performed IHC assay to determine the effect of miR-130b-3p in tubular formation *in vivo*. The results revealed that mice treated with miR-KD or the combination of miR-KD and OECM1-exo exhibited more tubular formation, whereas fewer blood vessels were found in mice treated with miR-KD or TSG101-KD (Figure 7F). Collectively, exosomal miR-130b-3p can promote tumor growth and tubular formation *in vivo* by targeting PTEN.

## Discussion

Oral squamous cell carcinoma has emerged as a primary cause of morbidity and mortality in patients with HNC (Chinn and Myers, 2015). Despite advances in surgical and adjuvant treatment, outcomes remain poor in patients with OSCC, which mainly resulted from late diagnosis, low survival rate, local recurrences, and poor prognosis (Li et al., 2017). Thus, there is an urgent need to explore the mechanism underlying the development and progression of OSCC, improving the treatment and management strategies for OSCC. In this study, we demonstrated that the protein level of PTEN, not mRNA, was reduced in OSCC, and miR-130b-3p was a primary negative regulator for post-transcriptional regulation of PTEN. Also, miR-130b-3p promoted angiogenesis in vascular cells through enhancing proliferation, migration, and ring formation. Meanwhile, OECM1-derived exosomes delivered miR-130b-3p to HUVECs to promote angiogenesis. Similarly, the effect of exosomal miR-130b-3p on tubular formation was also found in tumor xenograft mouse model.

Angiogenesis is a complex process of generating new blood vessels, which primarily takes place in development and reproduction (Wang et al., 2015). However, aberrant angiogenesis has been demonstrated to be involved in various pathologic processes, such as cancer, inflammation, ischemic, and immune diseases (Carmeliet, 2003). In cancers, since the angiogenetic process contributes to invasion and metastasis, tumor angiogenesis has emerged as a critical point to regulate cancer progression (Zhao and Adjei, 2015), thereby exploring antiangiogenic agents has become one of the primary therapeutic strategies for cancer treatment. In the present study, we identified that miR-130b-3p was a promotor for angiogenesis in OSCC *in vitro*, manifesting as enhancing HUVECs proliferation, migration, as well as ring formation and that the effect of miR-130b-3p on angiogenesis was abolished by knockdown of miR-130b-3p. Moreover, exosomal miR-130b-3p played a facilitative role in tumor growth and tubular formation *in vivo*. MiR-130b-3p is a multifunctional factor involved in several cancer types, including prostate cancer (Chen et al., 2015), endometrial cancer (Li et al., 2013), breast cancer (Shui et al., 2017), and bladder cancer (Cui et al., 2016). Regarding tumor angiogenesis, the upregulation of miR-130b promotes angiogenesis, cell proliferation, and epithelial-mesenchymal transition (EMT) in colorectal cancer (Colangelo et al., 2013), which is consistent with the observations in this study. However, the opposite role of miR-130b-3p in angiogenesis has been reported in prostate cancer (Mu et al., 2020), indicating that the role of miR-130b-3p in tubular formation displays a tumor-type-dependent pattern.

As a tumor suppressor, the protein level of PTEN was reduced in OSCC. Meanwhile, as the direct target gene of miR-130b-3p, PTEN functions as a key mediator in miR-130b-3p-induced angiogenesis in OSCC. Growing evidence shows that PI3K/PTEN signaling pathway exerts an important role in tumorigenesis and angiogenesis, in which the mutation of PI3K and the reduction of PTEN are widely seen in many types of solid tumors (He et al., 2016). In addition to angiogenesis, PTEN also plays a critical role in the progression of OSCC. Wang et al. reported that miR-655 inhibits cell invasion and proliferation in OSCC via targeting metadherin to influence PTEN/AKT signaling (Wang et al., 2018). Miyahara et al. (2018) demonstrated that PTEN allelic loss is a key factor in the transition from oral malignant lesions to OSCC. Furthermore, betulinic acid can increase radiosensitization in OSCC through upregulating PTEN associated with Sp1 sumoylation. Given the essential role in various biological activities, PTEN can be regarded as a promising biomarker to develop new therapeutic approaches for OSCC.

In the past decade, exosomes have drawn significant attention to modulating the development and progression of cancer due to their essential role in intercellular communication (Marleau et al., 2012). Exosomes can be internalized by target cells, in which the bioactive molecules, including miRNAs and enzymes, are transferred into recipient cells to modulating various cellular processes, such as apoptosis, proliferation, and autophagy (Taylor and Gercel-Taylor, 2011). To date, the role of exosomes in tumor progression has been uncovered in several cancers. For example, crbonic anhydrase 9-carrying exosomes derived from hypoxic renal cell carcinoma cells promote angiogenesis, which contributes to cancer progression (Horie et al., 2017). MiR-21-containing exosomes secreted from cisplatin-resistant OSCC cells can increase the cisplatin resistance of OSCC cells via targeting PTEN and PDCD4 (Liu et al., 2017). In addition, exosomal miR-29a-3p originated from OSCC can facilitate M2 subtype macrophage polarization (Cai et al., 2019). Collectively, exosome-mediated intercellular communication through miRNAs provides a new direction for cancer treatment.

## Conclusion

In conclusion, the results suggested that miR-130b-3p/PTEN promotes tubular formation in OSCC, suggesting their potential role in angiogenesis. Also, for future studies, the effect of miR-130b-3p on angiogenesis *in vivo* should be further investigated. Furthermore, because the OECM1 cell line was not comprehensively validated due to unexpected COVID lockdown, the findings of this study should be interpreted or applied with caution. This study demonstrated that exosomes are potential molecule delivery tools to regulate tumor progression *in vitro* and *in vivo*, indicating the significance of exosome-based strategy in clinical application.

## Data Availability Statement

The original contributions presented in the study are included in the article/Supplementary Material, further inquiries can be directed to the corresponding author/s.

## Ethics Statement

The studies involving human participants were reviewed and approved by Cangzhou Central Hospital. The patients/participants provided their written informed consent to participate in this study.

## Author Contributions

WY, YW, YC, and YG performed the material preparation, data collection, and analysis. QL and XW wrote the first draft of the manuscript. All authors contributed to the study conception and design, commented on previous versions of the manuscript, and read and approved the final manuscript.

## Conflict of Interest

The authors declare that the research was conducted in the absence of any commercial or financial relationships that could be construed as a potential conflict of interest.

## Footnotes

1 http://www.targetscan.org/

2 http://www.microrna.org/microrna/home.do
