# Supplementary material for: Exosomal miR-130b-3p Promotes Progression and Tubular Formation Through Targeting PTEN in Oral Squamous Cell Carcinoma
Source: Front Cell Dev Biol. 2021 Mar 22;9:616306. doi: 10.3389/fcell.2021.616306 (PMC8019696; doi:10.3389/fcell.2021.616306)
Supplement: Supplementary file 1 [file Data_Sheet_1.PDF]

Table 1. Patient characteristics

	N	Percentage (%)
**All cases**	20	
**Age(years)**		
**<60**	5	25
**≥ 60**	15	75
**Gender**		
**Male**	13	65
**Female**	7	35
**UICC stage**		
**I~II**	6	30
**III~IV**	14	70
**Grade**		
**Low**	8	40
**High**	12	60
**Tumor size(cm)**		
**< 3**	6	30
**≥ 3**	14	70
**Location of the tumors**		
**Border of tongue**	10	50
**Alveolar mucosa/gingiva/retromolar area**	6	30
**Floor of mouth/ventral tongue**	2	10
**Buccal mucosa/buccal sulcus**	2	10
**Therapy**		
**Radiotherapy**	11	55
**Chemotherapy**	9	45

**Surgery (R0-resection)**	20	100
**Median overall survival**		
**Months**	25.1	
**Range**	1-231	
