# Supplementary material for: Exosomal miR-130b-3p Promotes Progression and Tubular Formation Through Targeting PTEN in Oral Squamous Cell Carcinoma
Source: Front Cell Dev Biol. 2021 Mar 22;9:616306. doi: 10.3389/fcell.2021.616306 (PMC8019696; doi:10.3389/fcell.2021.616306)
Supplement: Supplementary file 2 [file Data_Sheet_2.PDF]

OECM-1 Human Oral Squamous Carcinoma Cell Line

Cancer Cell Line

Cat. # SCC180

Pack size: ≥1x10^6 viable cells/vial

Store in liquid nitrogen

FOR RESEARCH USE ONLY.
NOT FOR USE IN DIAGNOSTIC PROCEDURES.
NOT FOR HUMAN OR ANIMAL CONSUMPTION.

Data Sheet

Background

Squamous cell carcinoma (SCC) is a common cancer type, developing in epithelial cells. Nearly all cancers of the oral cavity are squamous cell carcinomas, and the high recurrence rate among oral SCCs make these challenging targets for therapies. Established models of this cancer type continue to yield significant insights into the properties and potential treatments of head and neck cancers.[1]

The OECM-1 human oral cavity squamous cell carcinoma cell line is a well-established model for squamous cell carcinoma. The OECM-1 cell line harbors a missense mutation in the *p53* tumor suppressor,[2] displays low EGFR expression,[3] and is tumorigenic in nude mice.[4] OECM-1 cells proliferate with a doubling time of 30-38 hours and are capable of anchorage-independent growth, forming spheroid colonies.[4] OECM-1 cells have been intensively characterized in the literature for morphology, biomarker expression and drug response,[5,6] and are widely utilized in studies of cancer cell signaling, epithelial-mesenchymal transition, metastasis and invasion, and cancer cell stemness.[7]

Source

The OECM-1 human oral cavity squamous cell carcinoma cell line was derived from surgical resection of a primary tumor of a Taiwanese male patient.[4]

Short tandem repeat (STR) Profile

D3S1358: 15, 18	D16S539: 10, 12
TH01: 9	CSF1PO: 12
D21S11: 29, 32.2	Penta D: 9, 10
D18S51: 16	vWA: 14, 16
Penta E: 20	D8S1179: 15
D5S818: 10, 11	TPOX: 8
D13S317: 10	FGA: 22, 27
D7S820: 8, 11	Amelogenin: X, Y

Cancer cell lines are inherently genetically unstable. Genetic instability may arise in the form of loss of heterozygosity of alleles at one or more genetic sites with increased passages.

Storage & Handling

OECM-1 human oral cavity squamous carcinoma cell line should be stored in liquid nitrogen. The cells can be cultured for at least 10 passages after initial thawing without significantly affecting the cell marker expression and functionality.

Quality Control Testing

- Each vial contains ≥ $1X10^6$ viable cells.
- Cells are tested negative for infectious diseases by a Human Essential CLEAR panel by Charles River Animal Diagnostic Services.
- Cells are verified to be of human origin and negative for inter-species contamination from rat, mouse, chinese hamster, Golden Syrian hamster, and non-human primate (NHP) as assessed by a Contamination CLEAR panel by Charles River Animal Diagnostic Services.
- Cells are negative for mycoplasma contamination.

Representative Data

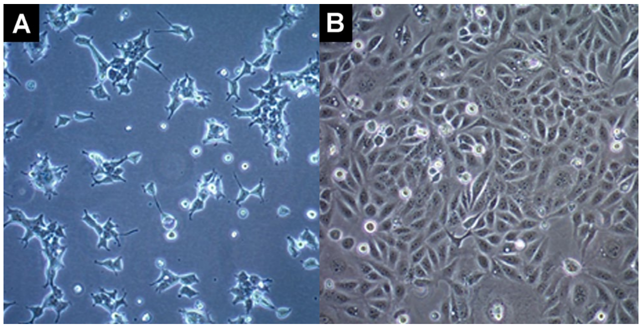

Figure 1. OECM-1 cells one (**A**, 10X magnification) and two (**B**, 10X magnification) days after thawing in a T75 flask.

References

1. Méry B et al., (2017) *Oral Oncol* 65: 51-56.
2. Lin SC et al., (2004) *J Oral Pathol Med* 33(2): 79-86.
3. Lee CH, Hung HW, Hung PH, Shieh YS (2010) *Mol Cancer* 9:64.
4. Yang CY, Meng CL (1994) *J Dent Res* 73(8): 1407-1415.
5. Huang GC, Liu SY, Lin MH, Kuo YY, Liu YC (2004) *Jpn J Clin Oncol* 34(9): 499-504.
6. Meng CL, Yang CY, Shen KL, Wong PY, Lee HK (1998) *Arch Oral Biol* 43(12): 979-986.
7. Chang CW et al., (2018) *Cell Death Dis* 9(2): 194.

Rev.1.0/2019-03-19/SCC180/VC
