# Supplementary figures and images for: Exosomal miR-130b-3p Promotes Progression and Tubular Formation Through Targeting PTEN in Oral Squamous Cell Carcinoma
Source: Front Cell Dev Biol. 2021 Mar 22;9:616306. doi: 10.3389/fcell.2021.616306 (PMC8019696; doi:10.3389/fcell.2021.616306)

## qRT-PCR primer amplification efficiencies

### Primer Amplification Efficiencies

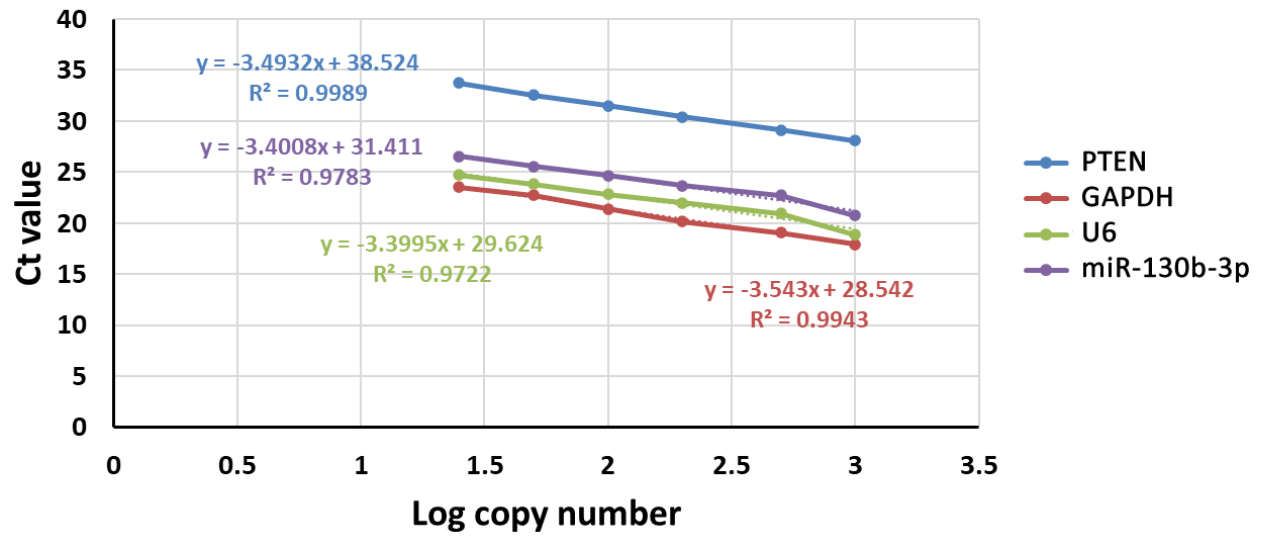

Supplement: Supplementary file 3 [file Data_Sheet_3.PDF]
